# Quantifying Actual and Perceived Inaccuracy When Estimating the Sugar, Energy Content and Portion Size of Foods

**DOI:** 10.3390/nu11102425

**Published:** 2019-10-11

**Authors:** Laura M. König, Katrin Ziesemer, Britta Renner

**Affiliations:** Department of Psychology, University of Konstanz, P.O. box 47, 78457 Konstanz, Germany; katrin.ziesemer@uni-konstanz.de (K.Z.); britta.renner@uni-konstanz.de (B.R.)

**Keywords:** sugar, estimation, energy, calories, weight, food, nutrition, portion size, accuracy, perception

## Abstract

In order to adhere to dietary guidelines and manage health risks, consumers need to be able to estimate with some accuracy the sugar and energy content of foods. The present study compared how well participants could estimate the sugar and energy content of foods, the weight of foods, and approximate portion size (using a hand measure estimation aid). The study had three aims. First, it aimed to investigate differences in accuracy across the four measures. Second, it aimed to examine whether these differences in accuracy between estimation measures were accurately perceived by the participants. Third, it aimed to test if estimation accuracy was related to food journaling experience, body-mass index or gender. One hundred and ninety-seven participants took part in an estimation task and filled in a questionnaire. While the participants were inaccurate when using all four estimation measures, inaccuracy was most pronounced for sugar content (*d*s ≥ 0.39), which was consistently overestimated by between 62.1% and 98.5% of the sample. None of the other measures showed a consistent pattern of under- or overestimation. Participants’ perceived accuracy did not match their actual accuracy (*r*s ≤ |0.20|, *p*s ≥ 0.005). Actual accuracy showed only marginal covariation with food journaling experience (*t*s ≤ 2.01, *p*s ≥ 0.049, *d*s ≤ 0.14), body-mass index (*r*s ≤ |0.15|, *p*s ≥ 0.041) or gender (*t*s ≤ 3.17, *p*s ≥ 0.002, *d*s ≤ 0.46). It is particularly challenging for consumers to estimate the sugar content of food, which might have negative consequences for health and well-being. Thus, more education about sugar content and misperceptions is needed to support consumers so that they can make healthy food choices.

## 1. Introduction

In 2015, the World Health Organization (WHO) published renewed guidelines for sugar intake [1]. As sugar intake is positively related to dental caries [2] and excess consumption of energy derived from sugar leads to overweight or obesity in both children and adults [3], the WHO recommends to limit daily sugar intake to 10% of total energy intake [1]. This recommendation is also reflected in the dietary guidelines of several national institutes such as the German Nutrition Society [4] and the Federal Centre for Nutrition in Germany, where the present study was conducted. The majority of the population consumes more sugar than recommended [5,6]. For instance, in Germany, men consume on average 124 g of sugar per day, while females consume on average 113 g per day [5], which is more than twice the recommended amount of 50 g per day [1,4]. Similarly, daily energy consumption exceeds current recommendations: In Germany, 36% of men and 31% of women consume more energy than recommended [5] which may lead to weight gain and, in the long term, overweight [7]. Thus, it is important to reduce sugar and energy consumed to reduce health risks.

Consumers need a better understanding of foods’ nutritional value, including sugar content, and energy content if they are to make choices that are in line with dietary recommendations. This is often promoted by nutritional labelling that provides numerical information on portion sizes as well as macronutrient and energy content. While this has proven effective in reducing the consumption of unhealthy foods and overall energy intake [8,9], there are many food choice situations in which nutritional labelling is not available, such as buying loose or unwrapped produce, or eating food that was prepared by someone else, e.g. in a restaurant. In these situations, consumers need to decide what and how much to eat based on their own perceptions of the food’s nutritional value and energy content. 

Previous research suggests that many consumers struggle to estimate accurately foods’ nutritional value: A study that specifically investigated estimations of sugar content showed that the majority of participants underestimated the sugar content of four out of six food items, although there was some variation in the under- and overestimation of sugar content between foods [10]. Other studies that investigated estimations of a meal’s energy content showed a general underestimation that increased with meal size [11,12,13,14,15,16]. Therefore, studies indicate that consumers often underestimate both the energy and sugar content of meals, which might lead to overconsumption and may subsequently have negative health consequences. 

However, as previous studies focused on estimating either energy or sugar content, it remains unclear if deviations from actual values are comparable between estimation measures, or whether one of the estimation measures might be more accurate and would thus be better suited for consumers to identify ‘healthy’ or ‘unhealthy’ foods. Therefore, the present study firstly aimed to assess how accurately consumers can estimate the sugar and energy content of different food items, and to compare these estimations with other measures commonly used to quantify food portion sizes. Specifically, the study took into account the foods’ weight (in grams) and the amount estimated by using a hand measure estimation aid, which typically indicates the number of handfuls a given food consists of ([17]; see also [18]), as these measures are commonly used in research, clinical practice [16], and available food tracking apps [19]. Accuracy was operationalised using mean levels, variability, and rank order of estimations, which allowed the study to investigate whether there is a general tendency to estimate inaccurately or if this is more pronounced for certain measures, thus testing generalisability of an under-/overestimation bias. 

Secondly, it examined consumers’ expected and perceived accuracy and its relation to objective estimation accuracy as determined by the estimation task. In this vein, it was investigated whether consumers are aware of their estimation (in-)accuracy and potential differences between the estimation measures correctly. Expected and perceived accuracy were assessed separately to be able to test whether evaluations changed due to the task, as participants might not have been familiar with certain estimation measures such as the hand measure. 

Thirdly, it aimed to assess the relationship between estimation accuracy and previous experience with food journaling (see also [13,14,20]) and gender [16,21] as potential influencing factors on estimation accuracy. Furthermore, body-mass index (BMI) was included as a potential outcome of chronic underestimation and subsequent overconsumption (c.f. [13,15,22]). In this vein, the study investigated if associations are comparable between estimation measures, thus representing generalizable relationships, or if they are specific to certain measures. 

## 2. Materials and Methods 

### 2.1. Sample

Sample size was determined using GPower 3.1 [23] based on the first aim of the study, which was to determine accuracy of estimations. In accordance with Block, Condon, Kleinman, Mullen, Linakis, Rifas-Shiman and Gillman [11], a small to medium effect (Cohen’s *d* = 0.3) was expected, which yielded an *n* of 147 for 95% power in a two-tailed one sample t-test. A convenience sample of *n* = 198 participants was recruited during a public open day at the University of Konstanz (“Lange Nacht der Wissenschaft”) in June 2018. One participant withdrew during the study, reducing the final sample to *n* = 197 (60.4% females, 39.1% males, 0.5% other). Participants were aged 19 to 74 (*M* = 37.46, *SD* = 15.50). Their BMI ranged from 16.07 to 35.08 (*M* = 23.37, *SD* = 3.12). Genders did not differ in age (*t* (192) = −0.75, *p* = 0.454), but in BMI (*t* (188) = −4.28, *p* < 0.001, *d* = 0.64; males: *M* = 24.53, *SD* = 2.92; females: *M* = 22.63, *SD* = 3.03).

### 2.2. Procedure

This study was conducted according to the guidelines laid down in the Declaration of Helsinki and all procedures involving human subjects were approved by the University of Konstanz ethics committee (no. 24/2018). Informed consent was obtained from all participants by them ticking a box in the online questionnaire. 

The study was advertised as a guessing game and was conducted in a booth in the university foyer. Participants who approached the booth were informed orally about the purpose of the study. They had to be at least 18 years old to be eligible for participation. If they agreed to take part, they were handed a tablet computer on which they filled in the questionnaire that guided them through the study. Participants were asked to complete the questionnaire individually and not talk to other participants during the study to ensure that estimates were not the result of a group effort. The questionnaire was administered using Qualtrics (Qualtrics, Provo, UT, USA, 2018). The first page displayed a brief explanation of the study and asked the participants to confirm that they understood the information, were aged 18 years or older, and wanted to participate. They were then introduced to the hand measure by reading a short, illustrated description showing what would be considered to be a handful or spoonful of different foods. They reported their expected accuracy during the estimation task before proceeding with the estimation task. Afterwards, they evaluated the estimation measures, reported their perceived accuracy and provided demographic and anthropometric information. Finally, they were thanked and allowed to choose one of the six foods as compensation. The participants were also given a leaflet containing a link to a webpage where the study results would be displayed the following day to ensure that they could not give other participants the correct answers. 

### 2.3. Materials and Measures 

#### 2.3.1. Food Items

Six foods were used in the study, comprising seven food items in total. Food items were chosen to be finger foods commonly consumed in Germany, to represent all the food categories contained in the German dietary guidelines (vegetables, fruit, dairy, protein sources, grains and starches, oils and fats, sugary foods; c.f. [17,24]), and to offer variations in weight, sugar and energy content. Furthermore, some food items were cut into small pieces (vegetables, fruit) while others were left in one piece (quiche, muffin) to vary the difficulty of using the hand measure. 

The savoury foods were a tomato-sheep cheese quiche, a cheese sandwich and vegetable sticks with a creamy yogurt-herb sauce, while sweet foods were chocolate mousse in a chocolate cup, fruit skewers, and a muffin (see Figure S1 in the Supplementary Material). 

To determine the foods’ weight, one piece of each food was disassembled to weigh the individual components separately. The weight of the individual components was entered into the software OptiDiet Basic version 5.1.0.042 (GOE mbH, Linden) using which sugar and energy content was determined based on the German Nutrient Database version 3.01 (Max Rubner-Institut, Karlsruhe, Germany). One of the experimenters measured how many of their handfuls of each food there were to establish the “correct solution”. These values are further referred to as *actual* amount/weight/sugar content/ energy content. 

#### 2.3.2. Estimation Task

In the estimation task, six food items were presented under a plastic sneeze guard (see Figure S2 in the Supplementary Material) and on pictures in the questionnaire. For each food, participants were asked to give estimates of four values and enter them in text boxes that allowed entering decimal numbers before proceeding to the next food. Firstly, they were asked to estimate the amount by using the hand measure [17]: they were asked to use their hand as reference and indicate how many handfuls of the item there were. For the creamy yogurt-herb sauce, participants were asked to use a tablespoon as a reference to indicate how many spoonfuls of sauce there were. Secondly, participants were asked to estimate the food’s weight in grams. Thirdly, they were asked to estimate the food’s sugar content (natural and/or added) in grams. Finally, they were asked to estimate the food’s energy content in kilocalories (kcal). These values are further referred to as *estimated* amount/ weight/ sugar content/ energy content. Foods were presented in a fixed order due to practical reasons of the set-up: (1) quiche, (2) cheese sandwich, (3) vegetable sticks and creamy yogurt-herb sauce, (4) chocolate mousse, (5) fruit skewers, (6) muffin. 

Additionally, for sugar, deviation in percent from the actual value were computed as follows:

$$\left(\frac{\mathrm{estimation}-\mathrm{actual}\;\mathrm{value}}{\mathrm{actual}\;\mathrm{value}}\right)\times 100$$

Based on this relative deviation, underestimation was defined as a relative deviation lower than 0, while overestimation was defined as a relative deviation greater than 0 (c.f. [10]). Estimations were accurate if relative deviation equalled 0.

#### 2.3.3. Expected and Perceived Accuracy 

Before the estimation task, participants were asked to indicate how accurate they thought their estimations would be (expected accuracy) on a Likert scale from (1) very accurate to (6) very inaccurate. After the estimation task, participants were asked to indicate how accurate they thought their estimations had been (perceived accuracy) on the same Likert scale. 

#### 2.3.4. Experience with Food Journaling 

Experience with food journaling was assessed using one item: “Have you ever recorded your food intake, e.g. using an app or a food diary?” Participants were able to reply either “yes” or “no”. 

#### 2.3.5. Demographic and Anthropometric Variables 

Participants were asked their age and gender (female, male, or other) and to report their height and weight, which were used to calculate BMI. 

### 2.4. Statistical Analysis 

Analyses were conducted with IBM SPSS 25 (released 2017). Missing values were 0.5% for the hand measure for the quiche and sandwich and the estimated sugar content of the creamy yogurt-herb sauce, 1% for age and weight, and 3% for height. 

Implausible values were checked for all estimations. For the estimated sugar content, a value was declared implausible if the estimated amount of sugar exceeded the estimated weight of the food item. For the hand measure, a value was declared implausible if it was equal to or greater than 10 (which is 10 times the amount of most food items presented and might have resulted from errors while handling the tablet computer, such as missing a decimal point). For the estimated amount in grams, a value was declared implausible if it exceeded 1000 g (which is approximately 6.6 times the amount of the heaviest food item presented). For the estimated energy content, a value was declared implausible if it exceeded 2500 kcal (which is the guideline daily amount of energy for men). Implausible values were replaced with a missing value. For the estimated amount of sugar, *n* = 2 (1%) entries were replaced for the quiche, *n* = 2 (1%) for the vegetable sticks, *n* = 19 (9.6%) for the creamy yogurt-herb sauce, *n* = 15 (7.6%) for the chocolate mousse, *n* = 6 (3%) for the fruit skewers, and *n* = 6 (3%) for the muffin. For the amount estimated using the hand measure, *n* = 2 entries (1%) were replaced for the quiche, *n* = 3 (1.5%) for the sandwich, *n* = 4 (2.0%) for the vegetable sticks, *n* = 8 (4.1%) for the creamy yogurt-herb sauce, *n* = 1 (0.5%) for the chocolate mousse, *n* = 3 (1.5%) for the fruit skewers, and *n* = 1 (0.5%) for the muffin. For the estimated weight, *n* = 2 (1%) entries were replaced for the quiche. For the estimated energy content, *n* = 1 (0.5%) entry was replaced for the sandwich and *n* = 2 (1%) were replaced for the creamy yogurt-herb sauce. 

Data were analysed using one sample or independent sample *t*-tests, within-subjects analyses of variance (ANOVAs), and bivariate correlations. 

## 3. Results 

### 3.1. Comparison between the Four Estimation Measures: Accuracy, Frequency, and Degree of Estimation Errors 

Table 1 depicts the actual values for the seven food items and the average estimation values provided by the participants. One-sample *t*-tests were conducted to test the accuracy of estimations, using the respective actual value as a reference. As Table 1 shows, 23 of the 28 estimations deviated significantly from the actual value. Overall, the largest effect sizes were found for the estimated sugar content (0.39 ≤ *d* ≤ 1.08).

As Table 2 shows, the percentage of participants who overestimated sugar content varied from 62.1% for the vegetable sticks to 98.5% for the quiche. Correspondingly, the proportion of participants underestimating sugar content was low, ranging from 1.5% for the quiche to 37.9% for the vegetable sticks. No consistent over- or underestimation was evident for the other three estimation measures (amount estimated by the hand measure, weight in grams, and energy content in kcal), although there was a general overall tendency to overestimate. 

In terms of the degree of estimation errors, the mean deviance in percent indicates that estimated sugar content had the largest mean overestimation for six of the seven food items (see Table 2). For example, 98.0% of the participants overestimated the sugar content of the sandwich, and estimations were more than ten times higher than the actual sugar content. While the degree of overestimation was higher than the degree of underestimation for all four estimation measures, the degree of overestimation was the highest for the perceived sugar content. Only for the chocolate mousse, overestimations were greater for the amount estimated with the hand measure (*M*hand measure = 360.00; *M*sugar content = 318.71). 

Covariances between the four different measures were calculated to further test if there was a general tendency to estimate inaccurately for certain estimation measures across food items. Deviance in percent within estimation measures were correlated between food items, indicating that there was a general trend to under-/ overestimate within a given estimation measure (estimated sugar content: 0.11 ≤ *r* ≤ 0.72; estimated amount: 0.02 ≤ *r* ≤ 0.58; estimated weight: 0.34 ≤ *r* ≤ 0.64; estimated energy content: 0.19 ≤ *r* ≤ 0.74). Effect sizes were predominantly medium to large [25]. All correlations are listed in Table S1 in the Supplementary Material. 

Deviation in percent within food items were also correlated, although effects were generally smaller than correlations within estimation measures (quiche: −0.04 ≤ *r* ≤ 0.27; sandwich: 0.08 ≤ *r* ≤ 0.47; vegetable sticks: 0.11 ≤ *r* ≤ 0.40; creamy yogurt-herb sauce: 0.07 ≤ *r* ≤ 0.44; chocolate mousse: −0.04 ≤ *r* ≤ 0.69; fruit skewers: 0.01 ≤ *r* ≤ 0.51; muffin: 0.00 ≤ *r* ≤ 0.57; see Table S2 in the Supplementary Material), indicating that there was a general tendency to under-/overestimate within a given food item. Overall, effects were largest for the correlations between estimated weight and sugar content (0.27 ≤ *r* ≤ 0.69). 

### 3.2. Expected and Perceived Accuracy of Estimations and Relation to Objective Accuracy 

Expected and perceived accuracy was compared between estimation measures using a within-subjects *Accuracy Estimate* × *Estimation Measure* ANOVA which yielded a significant main effect for estimation measure (*F* (2.25, 544.03) = 182.89, *p* < 0.001, *partial* η² = 0.48), indicating that accuracy estimations differed between estimation measures. This main effect was further qualified by a significant interaction (*F* (2.78, 544.03) = 9.73, *p* < 0.001, *partial* η² = 0.05). The interaction effect was followed up by simple main effects as recommended by Page, et al. [26], showing that a difference between measures existed both for the expected accuracy (before the estimation task; *F* (3, 588) = 106.85, *p* < 0.001, *partial* η² = 0.35) and the perceived accuracy (after the estimation task; *F* (3, 588) = 166.08. *p* < 0.001, *partial* η² = 0.46), although the effect for perceived accuracy (post-test) was stronger. Differences between estimation measures were followed up using paired comparisons. Both expected and perceived accuracy were highest for the hand measure (*M*_expected_ = 2.57, *SD*_expected_ = 0.88; *M*_perceived_ = 2.32, *SD*_perceived_ = 1.10), followed by the weight estimation (*M*_expected_ = 3.56, *SD*expected = 1.17; *M*perceived = 3.68, *SD*perceived = 1.16), the estimated sugar (*M*expected = 4.01, *SD*expected = 1.20; *M*perceived = 4.16, *SD*perceived = 1.21), and energy content (*M*expected = 3.95, *SD*_expected_ = 1.35; *M*_perceived_ = 4.19, *SD*_perceived_ = 1.31), which were the only two measures between which no significant difference was found (*p*s = 0.999; all other comparisons: *p*s ≤ 0.005). 

Expected and perceived accuracy, however, were only weakly linked to absolute deviation in percent (*r*s ≤ |0.20|, *p*s ≥ 0.005; see Table S3 in the Supplementary Material), as indicated by small effect sizes [25], and thus did not accurately reflect the observed inaccuracy in estimations. 

### 3.3. Relationships with Experience with Food Journaling, Gender and Body Mass Index (BMI) 

Absolute deviation was weakly linked to BMI (*r*s ≤ |0.15|, *p*s ≥ 0.041; see Table S1 in the Supplementary Material). Significant positive correlations only emerged for the amount of chocolate mousse (*r* (190) = 0.15, *p* = 0.045) and fruit (*r* (188) = 0.15, *p* = 0.042) estimated with the hand measure, indicating that estimations of participants with a higher BMI tended to deviate more strongly from the actual amount. When comparing participants who had previous experience with food journaling to unexperienced participants in independent samples t-tests (see Table S4 in the Supplementary Material), the only significant difference was for the estimated sugar content of the sandwich (*t* (66.13) = 2.01, *p* = 0.049, *d* = 0.35), and the found effect was small to medium [25]. Similarly, only small gender differences were found (see Table S5 in the Supplementary Material). Significant differences emerged for weight estimation in grams for the quiche (*t*(175.80) = 3.17, *p* = 0.002, *d* = 0.46) and the sandwich (*t*(191.98) = 2.84, *p* = .005, *d* = 0.40), for sugar estimation for the sandwich (*t*(176.66) = 2.03, *p* = 0.044, *d* = 0.27), and for estimating the amount of chocolate mousse using the hand measure (*t*(85.65) = −2.91, *p* = 0.005, *d* = 0.46). 

## 4. Discussion

The first aim of the present study was to compare how accurately subjects could quantify the amount and nutritional content of their food using four estimation measures. On average, participants’ estimations were inaccurate using all measures, but the magnitude and direction of incorrect estimations varied considerably across measures. Estimates of the foods’ sugar content showed the most pronounced deviations, and the sugar content of all food items was consistently overestimated. This general overestimation was not present in the other three measures, indicating that it might be specific to the estimation of sugar content. 

In the context of energy content estimation, Chernev and Chandon [27] suggested that people infer energy content from other cues such as macronutrient content when no unambiguous energy information is present. Interestingly, although the correlations between estimations of sugar and energy content indicated medium effects [25], overestimation of sugar content was not consistently reflected in an overestimation of energy content, and correlations between the two measures were only small to medium. Although estimating a food’s energy content requires aggregating information about multiple macronutrients, i.e. fat, protein and carbohydrates, deviation and variation were less pronounced than for the estimation of sugar content. Participants might have been more familiar with energy content because energy content is listed first on many nutrition labels on food packaging, either on top of a list of nutrients in a table format or at the left side on the guideline daily amount (GDA) nutrition labelling [28]. This might lead to a primacy effect, i.e. the information that is presented first is remembered best [29,30]. Furthermore, the GDA nutrition labelling also allows displaying only energy content on the front-of-pack label [28], which might further increase familiarity with the measure. Consequently, higher familiarity with energy content might have led to more accurate estimations. 

While sugar content was consistently overestimated in the present study, it was underestimated for the majority of foods in the study by Dallacker et al. [10]. This difference might at least partly be attributed to a difference in the estimation measure for sugar content: Dallacker et al. [10] asked participants to estimate sugar content in sugar cubes, while in the present study, sugar content was estimated in grams. These different methods of assessment might trigger differential cognitive representations. Sugar cubes represent units that can easily be counted, and are usually used in small quantities (e.g. one or two in a cup of coffee). As one sugar cube represents one unit, underestimation might be explained by the unit bias [31]: If a unit used for estimation is small, it might induce the expectation that a small amount of these units is sufficient, which subsequently lead to underestimation. Weight in grams, on the other hand, is a continuous measure without predefined units that is often used in larger quantities when related to sugar (e.g. 150 g sugar in a cake recipe). Furthermore, it has been shown that people tend to round to the next multiple of 10 or even 100 or their half-way points (for a summary, see Isaac and Schindler [32]), which might also be the case when estimating sugar content. This might contribute especially to overestimation when sugar content is comparably low due to small serving sizes, as was the case in the present study. Future research should compare different estimation measures specifically for sugar content to determine the most accurate measure and subsequently inform the communication of dietary recommendations and sugar content of foods. 

Secondly, the present study aimed to investigate the relationship between consumers’ expected and perceived estimation accuracy and their actual performance in order to determine if people are aware of their inability to accurately estimate food intake. Interestingly, the rank order of estimation inaccuracy was only partly reflected in the participants’ expected and perceived accuracy. Although participants both expected and perceived themselves to be least able to estimate accurately sugar content, participants did not expect to differ in how accurately they estimated sugar and energy content, indicating that they are not aware of their considerable tendency to overestimate sugar content. Sugar content might be a misleading cue for healthy food choices for people trying to follow dietary recommendations, and constant overestimation might lead to food being erroneously classified as less healthy, which could then lead to an unnecessary restraint of food intake, e.g. avoiding foods which are wrongly perceived as less healthy. Subsequently, this might increase the risk of developing an eating disorder [33] and reduce the enjoyment of eating and well-being [34,35,36].

Moreover, the significant interaction between expected and perceived accuracy indicates that accuracy ratings changed as a result of completing the estimation task. Differences between estimation measures were somewhat more pronounced after the estimation task, as indicated by a larger effect size. Moreover, comparison of means indicates that participants rated the hand measure to be more accurate after they completed the estimation task, while the other three estimation measures were rated to be less accurate after completing the estimation task. This indicates that by gaining more experience with the hand measure, participants were even more convinced of its accuracy. 

Thirdly, the present study aimed to investigate associations between estimation accuracy and experience of food journaling, gender and BMI, which were also investigated in previous studies on energy content and portion size estimation [13,14,15,16,20,21,22,37]. In line with previous studies comparing accuracy of energy content estimation between trained and untrained participants [13,20] and participants with low vs. high nutrition involvement [14], only weak associations were found between estimation accuracy and experience with food journaling. Relationships were comparable between estimation measures, indicating a generalizable missing link between experience and estimation accuracy. People who record their food intake, e.g. by using a food diary or an app, often do not receive feedback if their estimates are accurate. Since feedback is necessary to increase performance [38], they have no opportunity to adjust their estimations accordingly, and thus, no change occurs.

Due to potential differences in preoccupation with food and health knowledge [39,40,41], it could also be hypothesised that there is a gender difference in estimation accuracy. However, in the present research, there were only few notable gender differences, and these differences did not show a clear tendency for one gender to be more accurate than the other: for three of the significant comparisons, men were more accurate than women, while for one comparison, both genders were equally inaccurate but deviated in different directions. Similarly, only weak associations were found between estimation accuracy and BMI across estimation measures, again indicating a generalizable association and suggesting that estimation accuracy might not have a strong impact on body weight. Results of previous studies have been mixed, with two studies reporting no relationships between accuracy of calorie estimation and BMI [10,13], while another reported a positive relationship between inaccuracy and BMI [22]. The previous studies and the present one, however, analysed the relationship between estimation accuracy and BMI cross-sectionally, thus there is only limited evidence for the non-existence/existence of a causal effect, which would need to be investigated using longitudinal designs.

The present findings highlight that accurate quantification of energy or macronutrient content of foods is difficult for consumers. One potential avenue would be to provide more elaborate nutrition education in schools. However, research suggests that even when well educated about nutrition, providing accurate estimations remains difficult [13] and thus, numerical information such as grams of macronutrients or kilocalories might not be accessible cues for healthy foods. Alternative cues for healthy food choices, therefore, need to be identified that are more intuitively understood. For instance, sugar cubes could be used instead of sugar in grams to indicate sugar content on food packaging to provide more concrete information about sugar content [42]. Another avenue could be to promote meal colour variety as a cue for healthy food choices, which has been shown to be effective in previous studies [43,44]. In this vein, healthy food choices could be promoted without the need for promoting more accurate estimations. 

Generalisability of the findings might be limited due to missing information about the participants’ level of education and nutrition knowledge. As the study was conducted during a public engagement event at a university and one might speculate that the education level of the present sample might have been above average. However, as level of education is positively associated with nutrition knowledge [45,46,47] and health literacy [48], it can be suggested that the present study under- rather than overestimates the true effect. Furthermore, profession was not assessed. One could speculate that participants with a nutrition-related profession (e.g. dieticians) could have been more accurate in their estimations. Previous research has shown that dieticians show similar patterns of misestimations as regular consumers, albeit smaller [13]. Thus, not taking the profession into account might rather have led to under- rather than overestimation of the true effect. Future studies, therefore, should take level of education or other measures of socioeconomic status, nutrition knowledge and profession into account. Moreover, the present study did not take dietary restrictions, habitual food intake or current hunger levels into account, which have been related to portion size estimations in previous studies (e.g., Brunstrom, Rogers, Pothos, Calitri and Tapper [21], Brogden and Almiron-Roig [49]; see Almiron-Roig, Navas-Carretero, Emery and Martínez [16] for a review) and therefore might need to be considered again in future studies on nutrient content estimation. Finally, BMI was based on self-reported height and weight which might have potentially led to lower estimates of BMI, although based on previous research, it can be expected that rank order of BMI is still accurate due to very high correlations between self-reported and measured BMI [50]. 

It is usually seen as an advantage of the hand measure estimation aid that the size of the hand correlates with body size and, therefore, portion size estimations using the hand measure take differences in daily energy requirements into account [17]. In the present study, however, estimations of participants with especially large or small hands might have diverged from the “correct solution” due to differences to the standard hand (provided by a normal-weight adult female; length of 16.5 cm and breadth of 7.6 cm and thus comparable to the average female hand [51,52]) used to quantify the amounts which might have skewed the results. Still, estimations using the hand measure have been most accurate in comparison to the other three measures, which might suggest that differences in the size of participants’ hands might have only played a minor role.

Finally, the present study focused on the estimation of sugar and energy content. However, other macronutrients such as saturated fats are also related to the development of noncommunicable diseases such as cardiovascular diseases [53]. Therefore, it might be of interest to future studies to test whether the difficulties people experience when estimating sugar content also extend to fat content, and whether fat content is also generally underestimated. 

## 5. Conclusions

Estimating the sugar content of foods is very difficult for consumers, and the present study shows that this is even more difficult than estimating energy content. Although consumers perceived the estimation of the amount and weight of the food to be easier, they did not accurately represent the difference in accuracy between sugar and energy content, which might lead to erroneous choices of cues for healthy food choices. To enable consumers to adhere to dietary guidelines, future research needs to identify cues for healthy food choices that are easier to use and lead to higher accuracy. 

## Figures and Tables

**Table 1:** Estimation accuracy by food item and estimation measure.

Food Item	Actual Value (Ref.)	Estimated Value					
		M	SD	t	df	p	Cohen’s d
Quiche							
Sugar content (absolute, gram)	1.74	17.86	16.45	13.68	194	<0.001	0.98
Sugar content (% deviation)		926.38	945.65				
Amount (hand measure)	1 ^a^	1.0	0.37	−0.08	193	0.939	0.01
Weight (gram)	113	141.67	73.94	5.41	194	<0.001	0.39
Energy content (kcal)	372	269.53	201.63	−7.13	196	<0.001	0.51
Sandwich							
Sugar content (absolute, gram)	1.91	21.84	28.90	9.68	196	<0.001	0.70
Sugar content (% deviation)		1051.72	1512.17				
Amount (hand measure)	1 ^a^	1.13	0.48	3.79	193	<0.001	0.27
Weight (gram)	86	147.70	78.87	10.98	196	<0.001	0.78
Energy content (kcal)	298.6	238.67	132.45	−6.34	195	<0.001	0.45
Vegetable sticks							
Sugar content (absolute, gram)	3.43	8.58	13.29	5.41	194	<0.001	0.39
Sugar content (% deviation)		150.24	387.58				
Amount (hand measure)	1 ^a^	1.35	0.53	9.12	192	<0.001	0.66
Weight (gram)	88	96.71	65.75	1.86	196	0.064	0.13
Energy content (kcal)	21.5	54.21	46.54	9.87	196	<0.001	0.70
Creamy yogurt-herb sauce							
Sugar content (absolute, gram)	1.14	9.58	13.62	8.27	177	<0.001	0.62
Sugar content (% deviation)		492.30	794.35				
Amount (hand measure)	1 ^b^	2.13	1.22	12.70	188	<0.001	0.95
Weight (gram)	40.2	42.56	32.59	1.02	196	0.310	0.07
Energy content (kcal)	75.3	78.27	83.12	0.50	194	0.619	0.04
Chocolate mousse							
Sugar content (absolute, gram)	7.66	31.34	21.89	14.60	181	<0.001	1.08
Sugar content (% deviation)		309.15	285.76				
Amount (hand measure)	0.5 ^a^	0.86	1.22	4.10	195	<0.001	0.29
Weight (gram)	30.5	57.90	41.86	9.19	196	<0.001	0.65
Energy content (kcal)	142.93	245.61	170.80	8.44	196	<0.001	0.60
Fruit skewers							
Sugar content (absolute, gram)	5.11	27.06	24.44	12.42	190	<0.001	0.90
Sugar content (% deviation)		429.61	478.24				
Amount (hand measure)	1 ^a^	1.54	0.69	10.91	193	<0.001	0.78
Weight (gram)	60	84.19	53.03	6.40	196	<0.001	0.46
Energy content (kcal)	27.5	90.72	69.69	12.73	196	<0.001	0.91
Muffin							
Sugar content (absolute, gram)	19.4	44.52	32.65	10.63	190	<0.001	0.77
Sugar content (% deviation)		129.48	168.31				
Amount (hand measure)	1 ^a^	1.09	0.62	2.04	195	0.043	0.15
Weight (gram)	69	121.61	67.30	10.97	196	<0.001	0.78
Energy content (kcal)	249	243.86	150.24	−0.48	196	0.631	0.03

Note. ^a^ hand used as estimation aid; ^b^ table spoon used as estimation aid.

**Table 2:** Frequency (%) and degree (*M*, *SD*) of estimation errors by food type and estimation measure.

Food Item	Underestimation		Correct	Overestimation	
	N (%)	M ^1^ (SD)	N (%)	N (%)	M ^2^ (SD)
Sugar content (in grams)					
Quiche	3 (1.5)	−90.42 (16.59)	0 (0.0)	192 (98.5)	942.27 (944.35)
Sandwich	4 (2.0)	−92.15 (12.46)	0 (0.0)	193 (98.0)	1075.43 (1518.71)
Vegetable sticks	74 (37.9)	−60.92 (31.55)	0 (0.0)	121 (62.1)	279.37 (444.90)
Creamy yogurt-herb sauce	16 (9.0)	−37.70 (27.98)	0 (0.0)	162 (91.0)	544.65 (814.22)
Chocolate mousse	5 (2.7)	−29.50 (7.15)	0 (0.0)	177 (97.3)	318.71 (283.95)
Fruit skewers	18 (9.4)	−18.46 (27.06)	0 (0.0)	173 (90.6)	476.23 (478.94)
Muffin	29 (15.2)	−33.17 (17.88)	0 (0.0)	162 (84.8)	158.59 (166.60)
Overall	2 (1.0)	−28.24 (28.43)	0 (0.0)	195 (99.0)	522.32 (528.41)
Amount (hand measure)					
Quiche	40 (20.6)	−46.5 (10.20)	131 (67.5)	23 (11.9)	79.13 (39.96)
Sandwich	31 (16.0)	−43.72 (16.40)	116 (59.8)	47 (24.2)	82.45 (43.10)
Vegetable sticks	17 (8.8)	−41.71 (19.37)	84 (43.5)	92 (47.7)	80.44 (38.70)
Creamy yogurt-herb sauce	13 (6.9)	−56.92 (16.90)	19 (10.1)	157 (83.1)	140.73 (115.15)
Chocolate mousse	135 (68.9)	−60.94 (13.13)	46 (23.5)	15 (7.7)	360 (180.48)
Fruit skewers	13 (6.7)	−42.95 (24.68)	74 (38.1)	107 (55.2)	102.80 (54.21)
Muffin	35 (17.9)	−43.80 (16.68)	129 (65.8)	32 (16.3)	103.28 (106.60)
Overall	43 (21.9)	−18.55 (11.32)	3 (1.5)	150 (76.5)	45.27 (49.58)
Weight (in grams)					
Quiche	86 (44.1)	−32.35 (19.61)	0 (0.0)	109 (55.9)	70.91 (51.40)
Sandwich	43 (21.8)	−25.53 (21.01)	0 (0.0)	154 (78.2)	98.91 (85.15)
Vegetable sticks	105 (53.3)	−38.69 (21.69)	0 (0.0)	92 (46.7)	65.35 (75.18)
Creamy yogurt-herb sauce	124 (62.9)	−36.58 (28.43)	0 (0.0)	73 (37.1)	77.98 (90.18)
Chocolate mousse	67 (34.0)	−32.84 (20.66)	0 (0.0)	130 (66.0)	96.30 (111.83)
Fruit skewers	72 (36.5)	−33.84 (21.87)	14 (7.1)	111 (56.3)	93.50 (83.38)
Muffin	43 (21.8)	−33.54 (22.79)	0 (0.0)	154 (78.2)	106.90 (87.82)
Overall	57 (28.9)	−25.17 (19.45)	0 (0.0)	140 (71.1)	66.92 (57.79)
Energy content (in kcal)					
Quiche	167 (84.8)	−43.57 (21.80)	0 (0.0)	30 (15.2)	61.65 (86.18)
Sandwich	139 (70.9)	−43.01 (18.42)	0 (0.0)	57 (29.1)	35.87 (39.03)
Vegetable sticks	52 (26.4)	−37.03 (28.05)	0 (0.0)	145 (73.6)	219.97 (214.34)
Creamy yogurt-herb sauce	130 (66.7)	−44.41 (21.85)	0 (0.0)	65 (33.3)	100.63 (147.40)
Chocolate mousse	59 (29.9)	−28.38 (15.82)	0 (0.0)	138 (70.1)	114.68 (118.95)
Fruit skewers	17 (8.6)	−33.48 (19.10)	0 (0.0)	180 (91.4)	254.77 (251.17)
Muffin	111 (56.3)	−37.41 (20.63)	0 (0.0)	86 (43.7)	43.55 (64.09)
Overall	45 (22.8)	−23.75 (20.05)	0 (0.0)	152 (77.2)	82.77 (88.26)

Note. ^1^ Mean deviation in percent for participants who underestimated the food item. ^2^ Mean deviation in percent for participants who overestimated the food item.

## Supplementary Materials

The following are available online at https://www.mdpi.com/2072-6643/11/10/2425/s1, Figure S1. Food items used in the study, as depicted in the questionnaire. Top row, from left to right: quiche, sandwich, vegetable sticks and creamy yogurt-herb sauce. Bottom row, from left to right: chocolate mousse, fruit skewers, muffin, Figure S2. Food items placed under the plastic sneeze guard, as seen by participations during the estimation task, Table S1. Correlations (*N*) between deviations in percent within estimation measures and with BMI, Table S2. Correlations (*N*) between deviations in percent, Table S3. Correlations (*N*) between expected/ perceived accuracy and absolute deviation, Table S4. Comparison of deviation in percent for estimation measures between participants who were experienced and unexperienced with food journaling, Table S5. Comparison of deviation in percent for estimation measures between female and male participants.
