# Supplementary material for: Quantifying Actual and Perceived Inaccuracy When Estimating the Sugar, Energy Content and Portion Size of Foods
Source: Nutrients. 2019 Oct 11;11(10):2425. doi: 10.3390/nu11102425 (PMC6835963; doi:10.3390/nu11102425)
Supplement: Supplementary file 1 [file nutrients-11-02425-s001.pdf]

Supplementary Material

Figure S1. Food items used in the study, as depicted in the questionnaire. Top row, from left to right: quiche, sandwich, vegetable sticks and creamy yogurt-herb sauce. Bottom row, from left to right: chocolate mousse, fruit skewers, muffin.

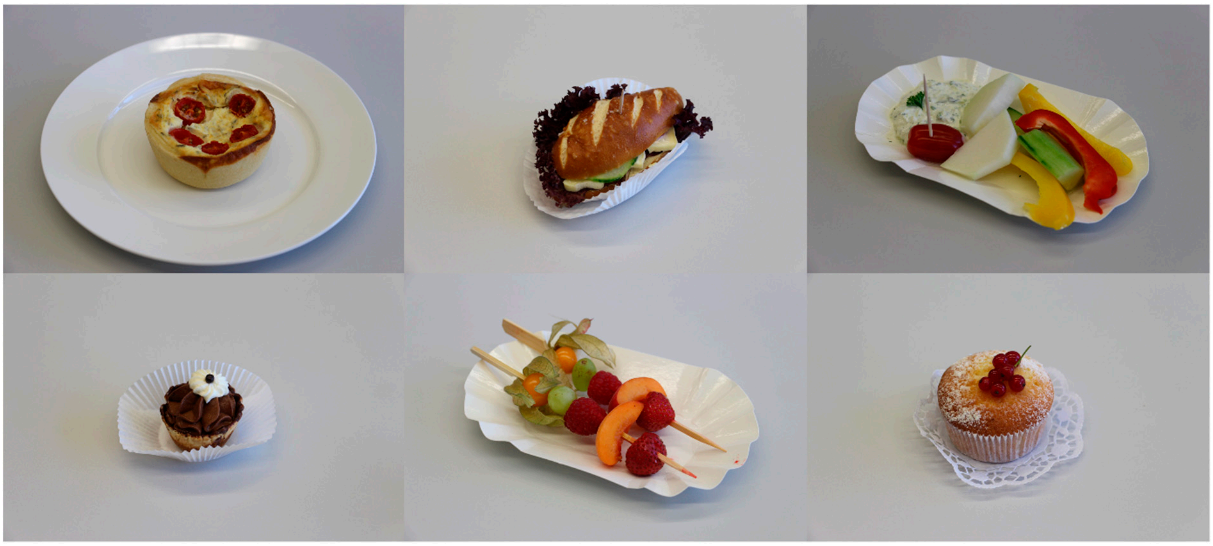

Figure S2. Food items placed under the plastic sneeze guard, as seen by participations during the estimation task.

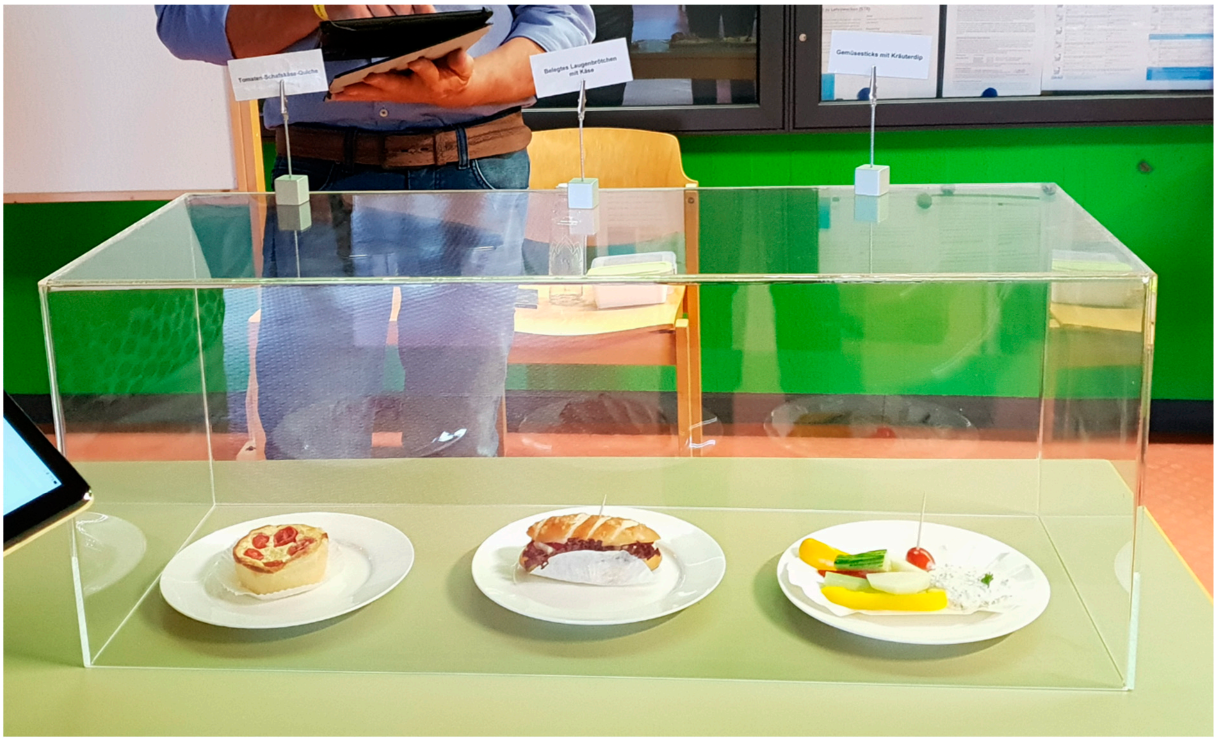

König, L. M., Ziesemer, K., & Renner, B. (2019). Quantifying actual and perceived inaccuracy when estimating the sugar, energy content and portion size of foods. *Nutrients*.

Table S1. Correlations (*N*) between deviations in percent within estimation measures and with BMI.

		2	3	4	5	6	7	8
Estimated sugar content								
1	Quiche	.72*** (195)	.39*** (194)	.11 (163)	.42*** (180	.27*** (190)	.58*** (189)	-.13 (189)
2	Sandwich		.57*** (195)	.39*** (178)	.44*** (182)	.38*** (191)	.52*** (191)	-.05 (191)
3	Vegetable sticks			.53*** (177)	.45*** (180)	.50*** (190)	.33*** (189)	.08 (189)
4	Creamy yogurt-herb sauce				.36*** (165)	.30*** (173)	.15* (173)	.06 (173)
5	Chocolate mousse					.42*** (180)	.60*** (182)	.02 (176)
6	Fruit skewers						.43*** (189)	.02 (185)
7	Muffin							-.06 (185)
8	BMI							
Estimated amount								
1	Quiche	.58*** (193)	.24** (191)	.16* (188)	.35*** (194)	.30*** (193)	.49*** (194)	.11 (188)
2	Sandwich		.26*** (191)	.07 (188)	.47*** (194)	.38*** (193)	.56*** (194)	.07 (188)
3	Vegetable sticks			.28*** (186)	.02 (193)	.38*** (191)	.35*** (193)	.05 (187)
4	Creamy yogurt-herb sauce				.14* (189)	.24** (187)	.17* (189)	.12 (183)
5	Chocolate mousse					.49*** (194)	.51*** (196)	.15* (190)
6	Fruit skewers						.46*** (194)	.15* (188)
7	Muffin							.02 (190)
8	BMI							
Estimated weight								
1	Quiche	.64*** (195)	.45*** (195)	.40*** (195)	.52*** (195)	.40*** (195)	.55*** (195)	-.12 (189)
2	Sandwich		.49*** (197)	.34*** (197)	.56*** (197)	.45*** (197)	.62*** (197)	-.10 (191)
3	Vegetable sticks			.50*** (197)	.46*** (197)	.55*** (197)	.45*** (197)	-.01 (191)

4	Creamy yogurt-herb sauce				.46*** (197)	.37*** (197)	.38*** (197)	.09 (191)
5	Chocolate mousse					.39*** (197)	.59*** (197)	-.09 (191)
6	Fruit skewers						.53*** (197)	-.10 (191)
7	Muffin							-.08 (191)
8	BMI							
Estimated energy content								
1	Quiche	.54*** (196)	.19** (197)	.28*** (195)	.36*** (197)	.28*** (197)	.35*** (197)	-.02 (191)
2	Sandwich		.40*** (196)	.43*** (194)	.53*** (196)	.46*** (196)	.51*** (196)	-.09 (190)
3	Vegetable sticks			.40*** (195)	.35*** (197)	.48*** (197)	.35*** (197)	-.02 (191)
4	Creamy yogurt-herb sauce				.54*** (195)	.34*** (195)	.53*** (195)	.00 (189)
5	Chocolate mousse					.50*** (197)	.74*** (197)	-.06 (191)
6	Fruit skewers						.53*** (197)	.01 (191)
7	Muffin							-.08 (191)
8	BMI							

Note. * $p < .05$; ** $p < .01$; *** $p < .001$

Table S2. Correlations (*N*) between deviations in percent.

		2	3	4
Quiche				
1	Sugar content	-.04 (192)	.27*** (193)	.16* (195)
2	Amount		.03 (192)	.10 (194)
3	Weight			.27*** (195)
4	Energy content			
Sandwich				
1	Sugar content	.16* (194)	.47*** (197)	.26*** (196)
2	Amount		.08 (194)	.08 (193)
3	Weight			.34*** (196)
4	Energy content			
Vegetable sticks				
1	Sugar content	.17* (191)	.30*** (195)	.35*** (195)
2	Amount		.18* (193)	.11 (193)
3	Weight			.40*** (197)
4	Energy content			
Creamy yogurt-herb sauce				
1	Sugar content	.08 (170)	.44*** (178)	.20** (176)
2	Amount		.09 (189)	.07 (187)
3	Weight			.29*** (195)
4	Energy content			
Chocolate mousse				
1	Sugar content	.01 (181)	.69*** (182)	.13 (182)
2	Amount		-.04 (196)	-.03 (196)
3	Weight			.27*** (197)
4	Energy content			
Fruit skewers				
1	Sugar content	.05 (188)	.51*** (191)	.27*** (191)
2	Amount		.01 (194)	.04 (194)
3	Weight			.37*** (197)
4	Energy content			
Muffin				
1	Sugar content	.05 (190)	.57*** (191)	.16* (191)
2	Amount		.01 (196)	.00 (196)
3	Weight			.31*** (197)
4	Energy content			

Note. * $p < .05$; ** $p < .01$; *** $p < .001$

Table S3. Correlations (*N*) between expected/ perceived accuracy and absolute deviation.

	Expected accuracy	Perceived accuracy
Estimated sugar content		
Quiche	.01 (195)	.06 (195)
Sandwich	-.06 (197)	.01 (197)
Vegetable sticks	-.04 (195)	-.05 (195)
Creamy yogurt-herb sauce	-.05 (178)	-.03 (178)
Chocolate mousse	-.01 (182)	-.04 (182)
Fruit skewers	.09 (191)	.07 (191)
Muffin	.11 (191)	.09 (191)
Estimated amount		
Quiche	-.04 (194)	.08 (194)
Sandwich	-.05 (194)	-.01 (194)
Vegetable sticks	-.06 (193)	.07 (193)
Creamy yogurt-herb sauce	.07 (189)	.01 (189)
Chocolate mousse	.10 (196)	.07 (196)
Fruit skewers	.07 (194)	.04 (194)
Muffin	.02 (196)	-.00 (196)
Estimated weight		
Quiche	.02 (195)	.12 (195)
Sandwich	.11 (197)	.11 (197)
Vegetable sticks	.08 (197)	.07 (197)
Creamy yogurt-herb sauce	.05 (197)	-.05 (197)
Chocolate mousse	.00 (197)	.01 (197)
Fruit skewers	.05 (197)	.10 (197)
Muffin	.12 (197)	.16* (197)
Estimated energy content		
Quiche	.20** (197)	.18* (197)
Sandwich	.17* (196)	.13 (196)
Vegetable sticks	.04 (197)	-.01 (197)
Creamy yogurt-herb sauce	.10 (195)	.11 (195)
Chocolate mousse	.09 (197)	.11 (197)
Fruit skewers	-.05 (197)	.01 (197)
Muffin	.13 (197)	.18* (197)

Note. * $p < .05$, ** $p < .01$

Table S4. Comparison of deviation in percent for estimation measures between participants who were experienced and unexperienced with food journaling.

Food item	Experienced		Unexperienced					
	M	*SD*	*M*	*SD*	*t*	*df*	*p*	Cohen's *d*
Quiche								
Estimated sugar content	1095.01	1206.48	854.99	805.17	1.63	193	.105	0.23
Estimated amount	4.66	40.91	-2.28	35.73	1.18	192	.238	0.18
Estimated weight	34.98	70.15	21.20	63.09	1.35	193	.177	0.21
Estimated energy content	-26.63	46.71	-27.94	57.26	0.15	195	.878	0.03
Sandwich								
Estimated sugar content	1491.53	2322.48	863.69	932.18	2.01	66.13[1]	.049	0.35
Estimated amount	19.91	56.08	10.03	43.57	1.32	192	.188	0.19
Estimated weight	86.17	105.15	65.58	84.99	1.45	195	.149	0.22
Estimated energy content	-14.31	54.97	-22.55	38.88	1.05	84.02[1]	.299	0.17
Vegetable sticks								
Estimated sugar content	185.47	475.78	134.95	343.27	0.84	193	.405	0.12
Estimated amount	40.02	61.84	32.37	48.47	0.92	191	.358	0.14
Estimated weight	10.84	60.10	9.49	80.35	0.12	195	.908	0.02
Estimated energy content	160.23	238.88	148.67	206.96	0.34	195	.732	0.05
Creamy yogurt-herb sauce								
Estimated sugar content	626.58	1224.09	433.83	500.99	1.12	60.86[1]	.268	0.21
Estimated amount	106.70	124.00	115.64	121.91	-0.46	187	.647	0.07
Estimated weight	6.81	89.12	5.47	77.70	0.11	195	.916	0.02
Estimated energy content	1.94	103.93	4.81	113.43	-0.17	193	.868	0.06
Chocolate mousse								
Estimated sugar content	333.23	282.65	298.99	287.56	.74	180	.462	0.16

Estimated amount	-34.34	79.36	-5.84	135.17	-1.51	194	-133	0.26
Estimated weight	45.99	77.19	55.11	121.72	-0.53	195	.596	0.09
Estimated energy content	56.21	108.83	78.52	123.55	-1.20	195	.231	0.19
Fruit skewers								
Estimated sugar content	495.86	539.93	400.71	447.84	1.27	189	.207	0.19
Estimated amount	49.91	54.70	55.45	73.91	-0.51	192	.610	0.09
Estimated weight	56.07	102.48	33.58	81.11	1.64	195	.102	0.24
Estimated energy content	237.87	261.95	226.48	250.60	0.29	195	.773	0.04
Muffin								
Estimated sugar content	163.34	193.31	115.07	155.03	1.83	189	.070	0.28
Estimated amount	18.56	87.29	4.94	47.33	1.13	73.11[1]	.263	0.19
Estimated weight	94.42	94.96	68.47	97.93	1.72	195	.087	0.27
Estimated energy content	-0.76	50.65	-2.63	64.20	0.20	195	.843	0.03

Note. [1] Corrected for heterogeneity of variances, as indicated by the Levene test.

Table S5. Comparison of deviation in percent for estimation measures between female and male participants.

Food item	Female		Male					
	M	*SD*	*M*	*SD*	*t*	*df*	*p*	Cohen's *d*
Quiche								
Estimated sugar content	991.71	1071.72	825.51	715.91	1.30	191.95[1]	.197	0.18
Estimated amount	1.31	35.85	-2.60	40.04	0.71	191	.481	0.10
Estimated weight	36.90	67.10	7.76	59.48	3.17	175.80[1]	.002	0.46
Estimated energy content	-23.74	58.36	-34.86	45.29	1.42	194	.158	0.21
Sandwich								
Estimated sugar content	1204.32	1818.35	817.25	819.31	2.03	176.66[1]	.044	0.27
Estimated amount	12.30	37.96	14.24	60.47	-0.25	111.29[1]	.804	0.04
Estimated weight	84.49	99.72	49.74	71.37	2.84	191.98[1]	.005	0.40
Estimated energy content	-18.80	39.72	-23.16	50.07	0.68	193	.501	0.10
Vegetable sticks								
Estimated sugar content	127.68	376.35	184.72	406.91	-1.00	192	.320	0.15
Estimated amount	38.66	49.80	28.63	57.34	1.28	190	.203	0.19
Estimated weight	7.88	79.02	13.27	68.40	-0.49	194	.624	0.07
Estimated energy content	153.16	213.32	148.99	223.56	0.13	194	.896	0.02
Creamy yogurt-herb sauce								
Estimated sugar content	414.20	701.53	607.23	907.71	-1.59	175	.113	0.24
Estimated amount	110.40	116.67	115.81	131.57	-0.30	186	.768	0.04
Estimated weight	1.35	86.29	12.94	72.82	0.98	194	.331	0.15
Estimated energy content	-7.69	106.80	22.00	114.86	-1.83	192	.069	0.27
Chocolate mousse								
Estimated sugar content	312.02	273.98	305.23	305.68	0.16	179	.876	0.02
Estimated amount	-38.10	58.27	22.46	175.60	-2.91	85.65[1]	.005	0.46
Estimated weight	57.67	116.03	44.46	101.41	0.82	194	.415	0.12

Estimated energy content	69.91	111.03	75.24	132.87	-0.30	194	.762	0.04
Fruit skewers								
Estimated sugar content	414.47	464.05	459.28	499.98	-0.63	188	.528	0.09
Estimated amount	54.83	57.72	52.96	83.73	0.17	118.60[1]	.865	0.03
Estimated weight	40.59	92.27	40.63	83.00	-0.00	194	.998	0.00
Estimated energy content	215.75	231.31	253.44	285.44	-1.02	194	.311	0.15
Muffin								
Estimated sugar content	125.07	183.21	136.91	145.60	-0.47	188	.636	0.07
Estimated amount	6.05	35.05	13.84	89.92	-0.72	89.72[1]	.473	0.11
Estimated weight	79.91	104.55	71.37	86.43	0.60	194	.552	0.09
Estimated energy content	-6.28	47.32	4.67	76.39	-1.24	194	.217	0.17

Note. [1] Corrected for heterogeneity of variances, as indicated by the Levene test.
